# Endoscopic Marsupialization of Frontal Sinus Cholesterol Granuloma via a Draf IIb Approach: A Case Report

**DOI:** 10.7759/cureus.116389

**Published:** 2026-09-16

**Authors:** Yuichi Teranishi, Kenta Uemura, Kazuki Amesara, Chiaki Nakahama, Yumi Takemiya

**Affiliations:** 1 Otolaryngology and Head and Neck Surgery, Osaka Metropolitan University, Osaka, JPN; 2 Otorhinolaryngology and Pediatric Otorhinolaryngology, Yodogawa Christian Hospital, Osaka, JPN; 3 Otorhinolaryngology and Head and Neck Surgery, Osaka City General Hospital, Osaka, JPN; 4 Otorhinolaryngology, Tane General Hospital, Osaka, JPN

**Keywords:** draf iib, endoscopic marsupialization, endoscopic sinus surgery, frontal sinus cholesterol granuloma, frontal sinusotomy

## Abstract

Frontal sinus cholesterol granuloma (CG) is a rare, non-neoplastic inflammatory lesion that has traditionally been managed by open surgical approaches. A 48-year-old man presented with a one-year history of localized swelling of the left forehead. Computed tomography demonstrated two expansile lesions in the left frontal sinus. The larger lateral lesion was surrounded by diffusely thinned bone, with focal defects in the anterior frontal sinus wall and orbital roof. It protruded through these defects into the orbit and displaced the orbital contents inferiorly. Magnetic resonance imaging showed predominantly high signal intensity on both T1- and T2-weighted images with minimal contrast enhancement, leading to a preoperative suspicion of CG; the smaller medial lesion was considered a mucocele. Endoscopic sinus surgery was performed. The medial lesion and lateral CG cavity were marsupialized, and a left Draf IIb frontal sinusotomy provided adequate access to the lateral frontal sinus. The CG cavity contained yellow-brown muddy fluid admixed with abundant yellow granular material. After irrigation and removal of the contents, complete excision of the cyst wall was not attempted. Exposed bone was covered with a superior lateral anterior pedicle flap and a middle turbinate-based mucosal flap, and a Penrose drain was positioned to maintain patency and permit postoperative irrigation. Histopathological examination confirmed CG. The drain was removed three months after surgery. The frontal sinusotomy and marsupialized opening remained patent, with no evidence of recurrence during 21 months of follow-up. Endoscopic marsupialization is a useful, minimally invasive treatment option for frontal sinus CG when adequate access can be achieved.

## Introduction

Cholesterol granuloma (CG) is a granulomatous lesion caused by a foreign-body reaction to deposited cholesterol crystals. It most commonly occurs in the temporal bone, particularly the petrous apex and middle ear, whereas involvement of the paranasal sinuses is rare [1,2]. Surgical treatment is the mainstay of management, and frontal sinus lesions have historically been treated predominantly through open approaches [1]. With advances in endoscopic sinus surgery (ESS), endoscopic treatment of frontal sinus CG has been reported [3]; however, published cases remain limited, and no consensus has been established regarding the optimal surgical approach. We report a case of frontal sinus CG with lateral extension that was successfully treated with endoscopic marsupialization via a Draf IIb approach.

This case was previously presented at the 17th Japan-Taiwan Conference on Otolaryngology-Head and Neck Surgery on December 6, 2025, and at the 35th Annual Meeting of the Japan Society for Head and Neck Surgery on February 6, 2026.

## Case presentation

A 48-year-old man presented with a one-year history of swelling of the left forehead. His medical history was notable for previous cholecystectomy, and his family history was unremarkable. He first visited a local otolaryngology clinic, where a frontal sinus tumor was suspected, and was referred to our department for further evaluation and treatment. He had no history of facial trauma or sinonasal surgery. Physical examination revealed a hard, localized swelling of the left forehead above the left eyebrow. Nasal endoscopy showed edematous mucosa and narrowing of the left middle meatus without purulent discharge or other significant abnormalities.

Computed tomography (CT) of the paranasal sinuses demonstrated two expansile soft-tissue-attenuation lesions in the left frontal sinus: a large lateral lesion and a smaller medial lesion. The bony walls surrounding the lateral lesion were diffusely thinned, with focal defects extending from the anterior frontal sinus wall to the orbital roof. The lesion protruded into the orbit through the bony defects and displaced the orbital contents inferiorly (Figure 1).

**Figure 1:**
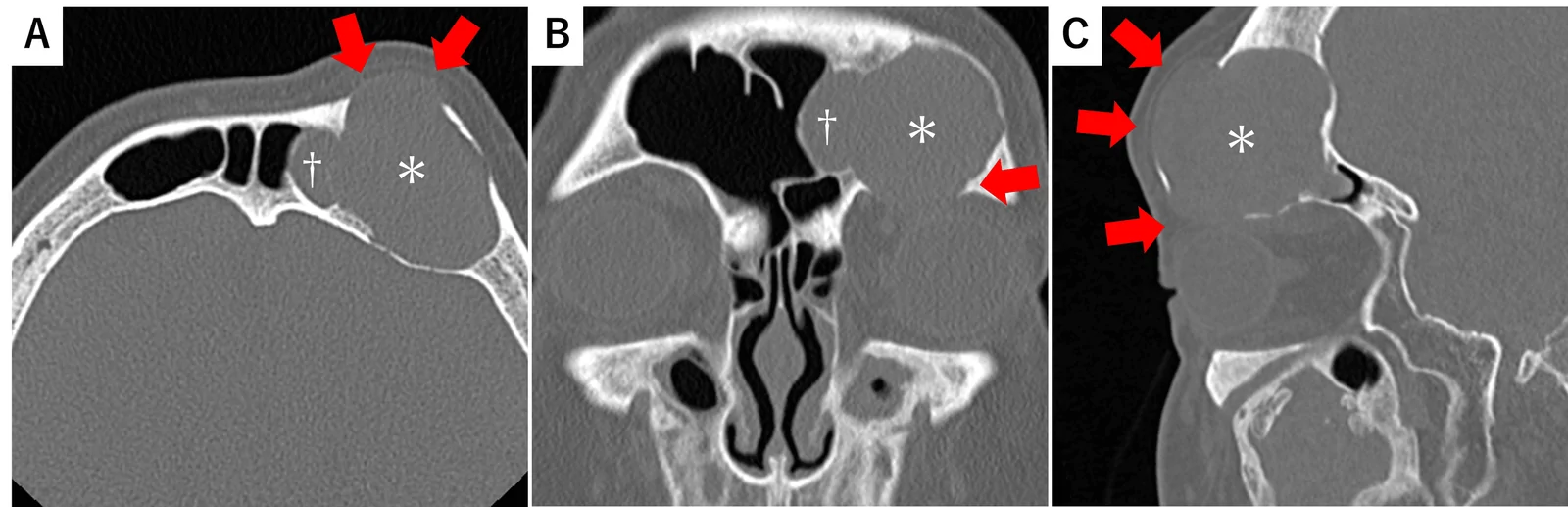
Preoperative computed tomography findings (A-C) Axial, coronal, and sagittal computed tomography images, respectively, demonstrated two lesions in the left frontal sinus. The bony walls surrounding the lateral lesion were diffusely thinned, with focal defects extending from the anterior frontal sinus wall to the orbital roof (arrows). The lesion protruded into the orbit through the bony defects and displaced the orbital contents inferiorly. ^*^lateral cholesterol granuloma lesion ^†^medial mucocele lesion

Magnetic resonance imaging (MRI) showed a well-circumscribed lateral lesion that was predominantly hyperintense on both T1- and T2-weighted images. A posterior portion of the lesion was mildly hyperintense on T1-weighted images and hypointense on T2-weighted images, suggesting hemorrhagic changes in the contents. The medial lesion was hypointense on T1-weighted images and hyperintense on T2-weighted images. Both lesions showed minimal enhancement after gadolinium administration (Figure 2). Based on these findings, the lateral lesion was suspected to be CG, whereas the smaller medial lesion was considered a mucocele, and surgical treatment was planned.

**Figure 2:**
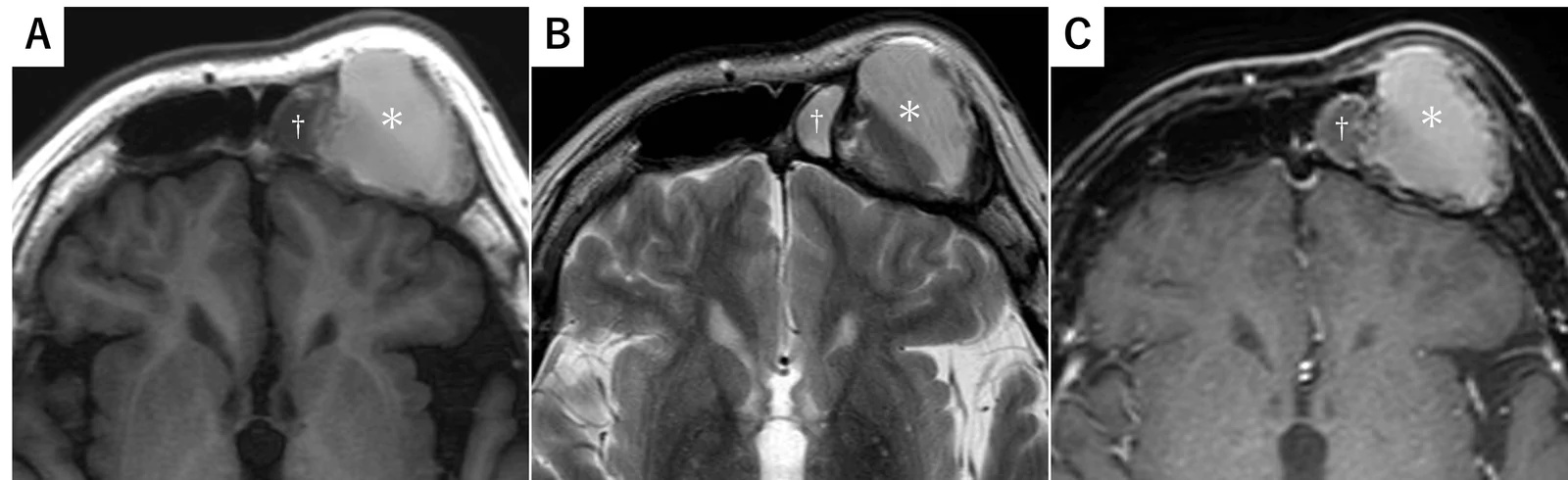
Preoperative magnetic resonance imaging findings (A) On the T1-weighted image, the lateral lesion was predominantly hyperintense, with a mildly hyperintense posterior portion, whereas the medial lesion was hypointense. (B) On the T2-weighted image, the lateral lesion was predominantly hyperintense, with a hypointense posterior portion, whereas the medial lesion was hyperintense. (C) On the contrast-enhanced fat-suppressed T1-weighted image, both lesions showed minimal enhancement. ^*^lateral cholesterol granuloma lesion ^†^medial mucocele lesion

ESS was performed under general anesthesia. The frontal sinus was first approached through the left middle meatus, and the presumed medial mucocele was marsupialized. A yellowish lesion suspected to represent CG was then identified laterally (Figure 3A). Upon opening the lesion, yellow-brown muddy fluid containing abundant yellow granular material was encountered. To enlarge the opening and allow manipulation within the cavity, a left Draf IIb frontal sinusotomy was performed (Figure 3B). A superior lateral anterior pedicle (SLAP) flap was elevated and preserved [4]. After drilling the frontal beak and frontal sinus floor, the lesion was marsupialized more widely (Figure 3C), and the cavity was inspected using a 70-degree angled endoscope (Figure 3D). The cavity was irrigated with saline using a Hydrodebrider System (Medtronic), and the contents were removed. A portion of the cyst wall was submitted for histopathological examination. Complete excision of the cyst wall was not attempted. Exposed bone was covered with two local pedicled mucosal flaps: the SLAP flap and a middle turbinate-based mucosal flap. The SLAP flap covered the anterior aspect of the frontal sinusotomy, whereas the middle turbinate-based flap covered the exposed medial bony surface. A Penrose drain was placed through the marsupialized opening. One end was positioned in the CG cavity and the other in the maxillary sinus; this configuration maintained the drain in position without suture fixation (Figure 3E). The operative time was three hours and 47 minutes, and blood loss was minimal.

**Figure 3:**
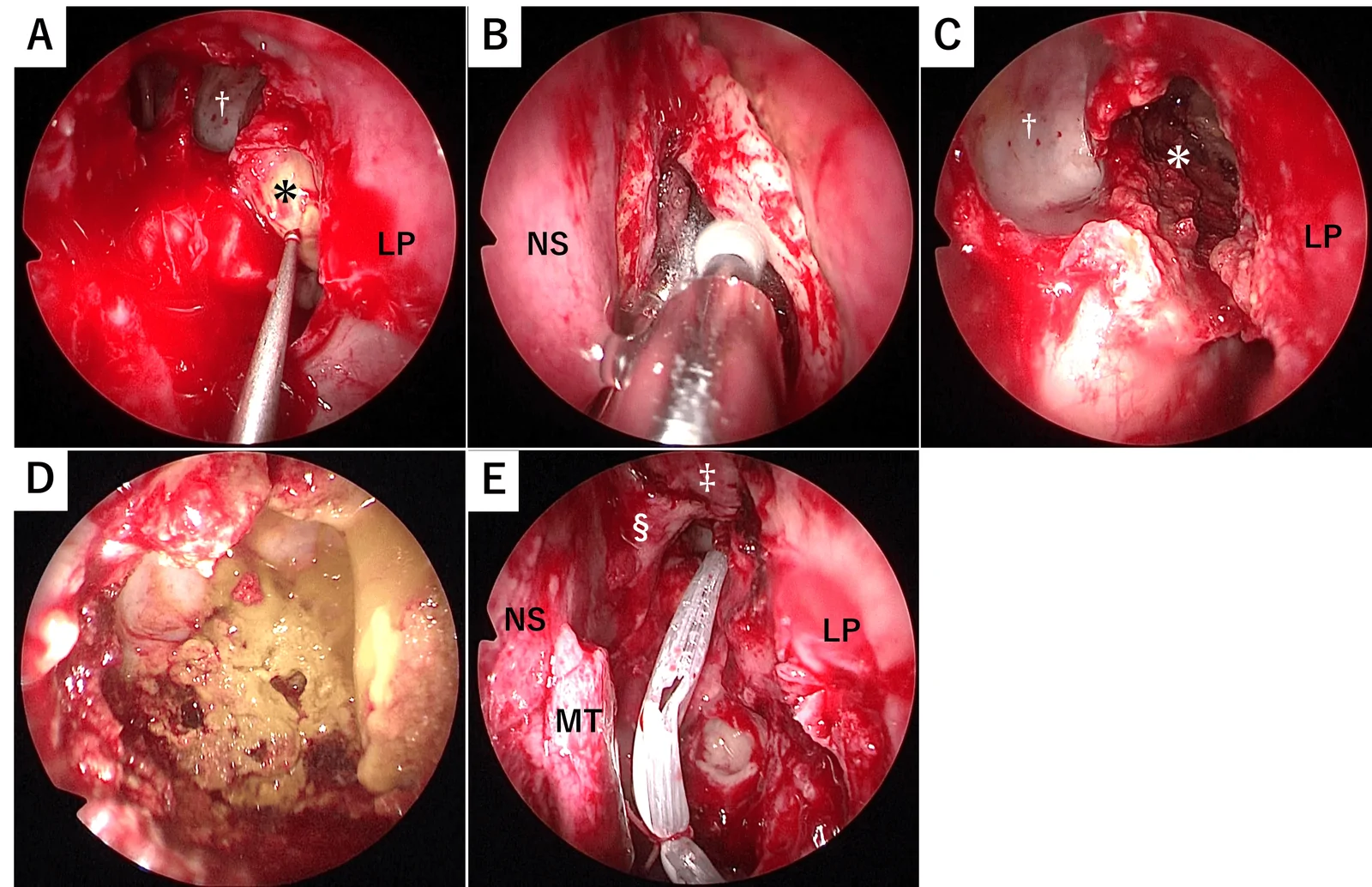
Intraoperative findings (A) A yellowish cholesterol granuloma lesion was identified in the lateral frontal sinus. The asterisk indicates the cholesterol granuloma lesion, and the dagger indicates the presumed medial mucocele after marsupialization. (B) A Draf IIb frontal sinusotomy was performed by drilling the frontal beak and frontal sinus floor. (C) The cholesterol granuloma lesion was marsupialized to create an adequate drainage pathway. The asterisk indicates the marsupialized cholesterol granuloma cavity, and the dagger indicates the marsupialized medial presumed mucocele. (D) The cholesterol granuloma cavity was examined using a 70-degree angled endoscope. Yellow-brown muddy fluid containing abundant yellow granular material was observed within the cavity. (E) Exposed bone was covered with local mucosal flaps, and a Penrose drain was placed through the marsupialized opening. The double dagger indicates the superior lateral anterior pedicle flap, and the section sign indicates the middle turbinate-based mucosal flap. LP, lamina papyracea; MT, middle turbinate; NS, nasal septum

Histopathological examination demonstrated granulomatous inflammation with cholesterol clefts and hyalinized fibrous tissue, confirming the diagnosis of CG (Figure 4).

**Figure 4:**
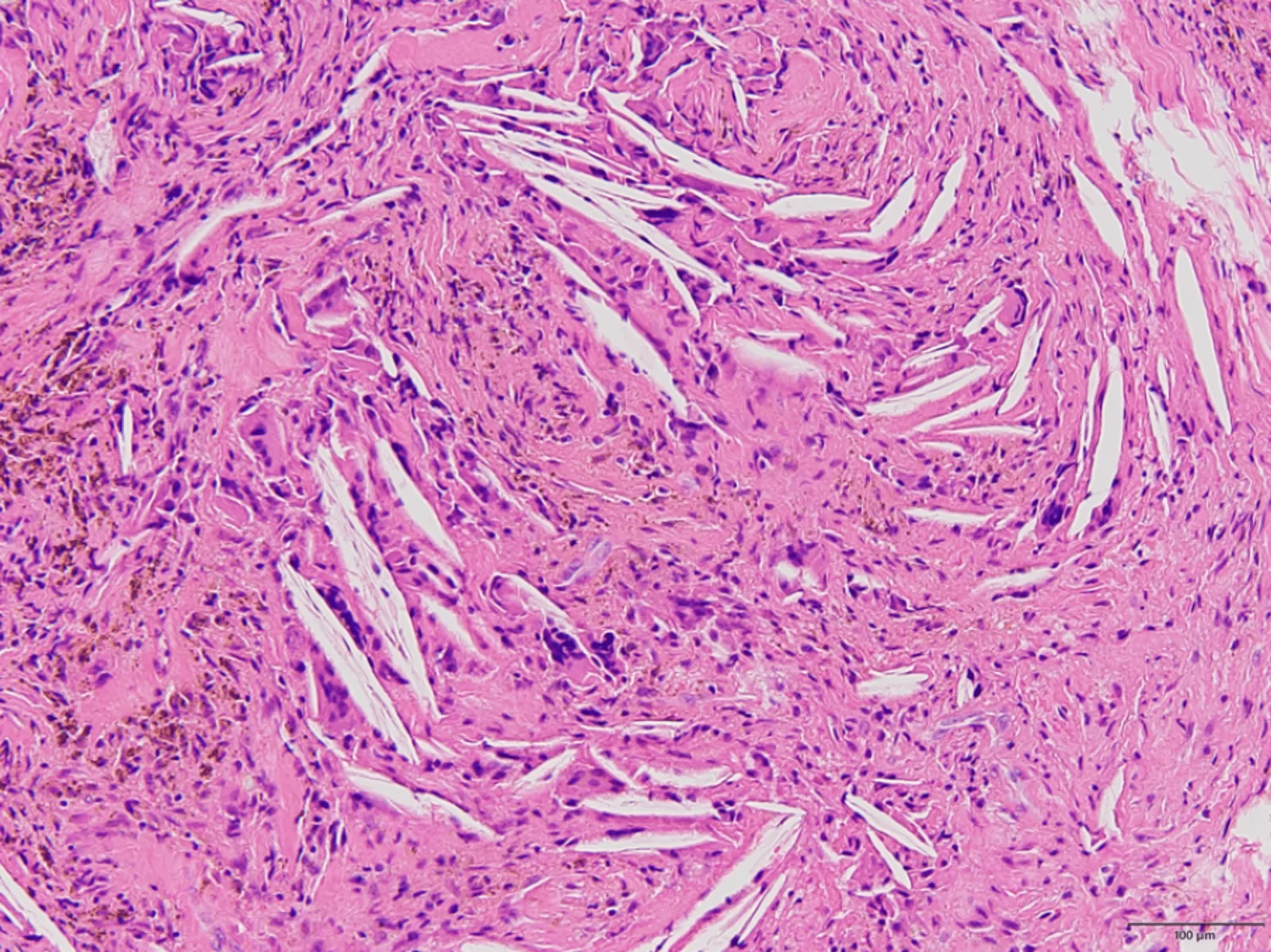
Histopathological findings Hematoxylin and eosin staining (original magnification, ×100) demonstrated cholesterol clefts, granulomatous inflammation, and hyalinized fibrous tissue, consistent with cholesterol granuloma.

The postoperative course was uneventful, without infection, bleeding, or other complications. At outpatient visits, the CG cavity was irrigated with saline through the Penrose drain, which was removed three months after surgery. The forehead swelling improved. Endoscopic examination and CT confirmed that both the frontal sinusotomy and the marsupialized opening remained patent (Figure 5 and Figure 6), and no recurrence was observed through 21 months of follow-up.

**Figure 5:**
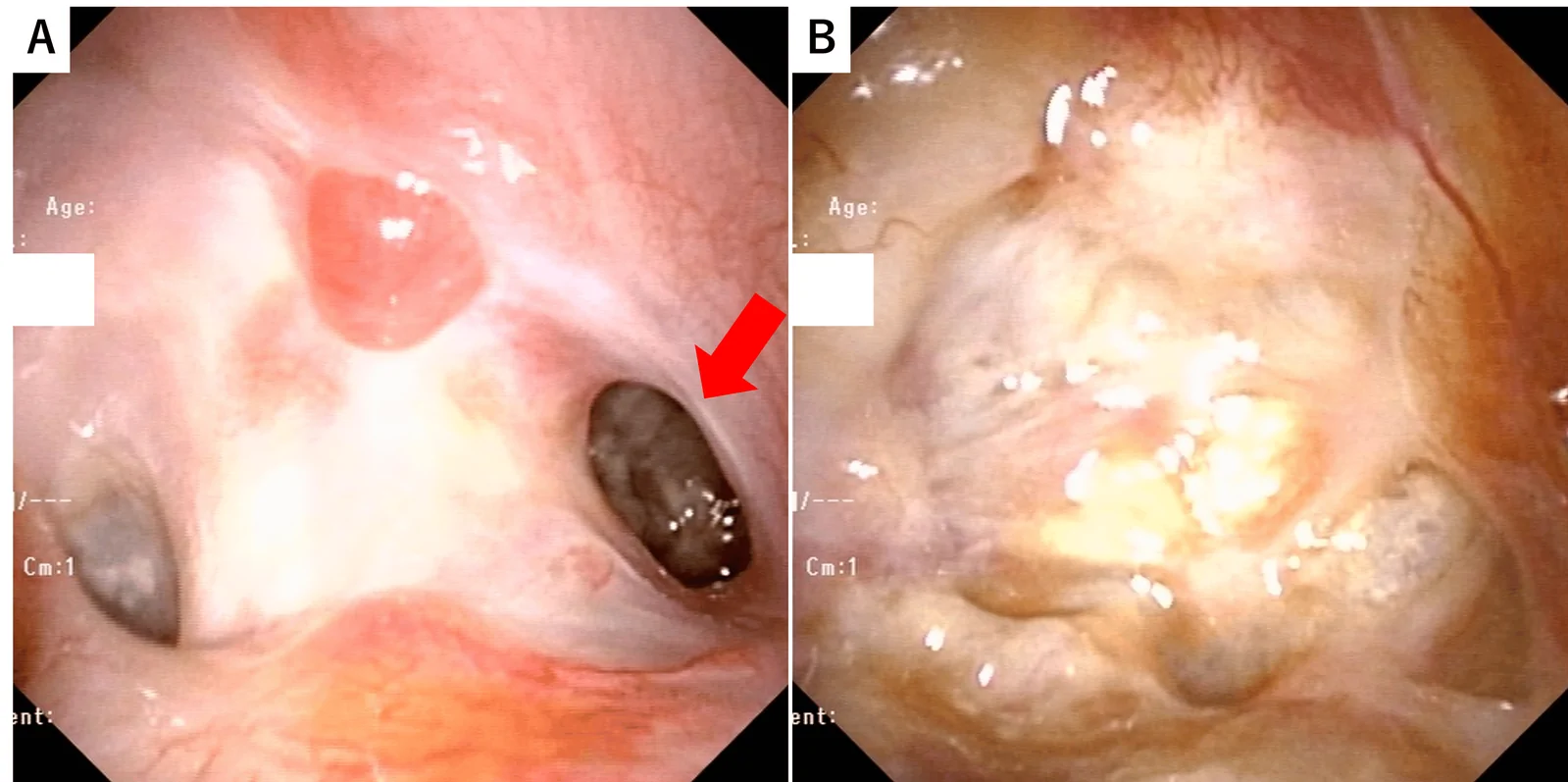
Endoscopic findings five months after surgery (A) The marsupialized opening of the cholesterol granuloma cavity in the left frontal sinus remained patent (arrow). (B) The cavity contained no retained material and was well epithelialized.

**Figure 6:**
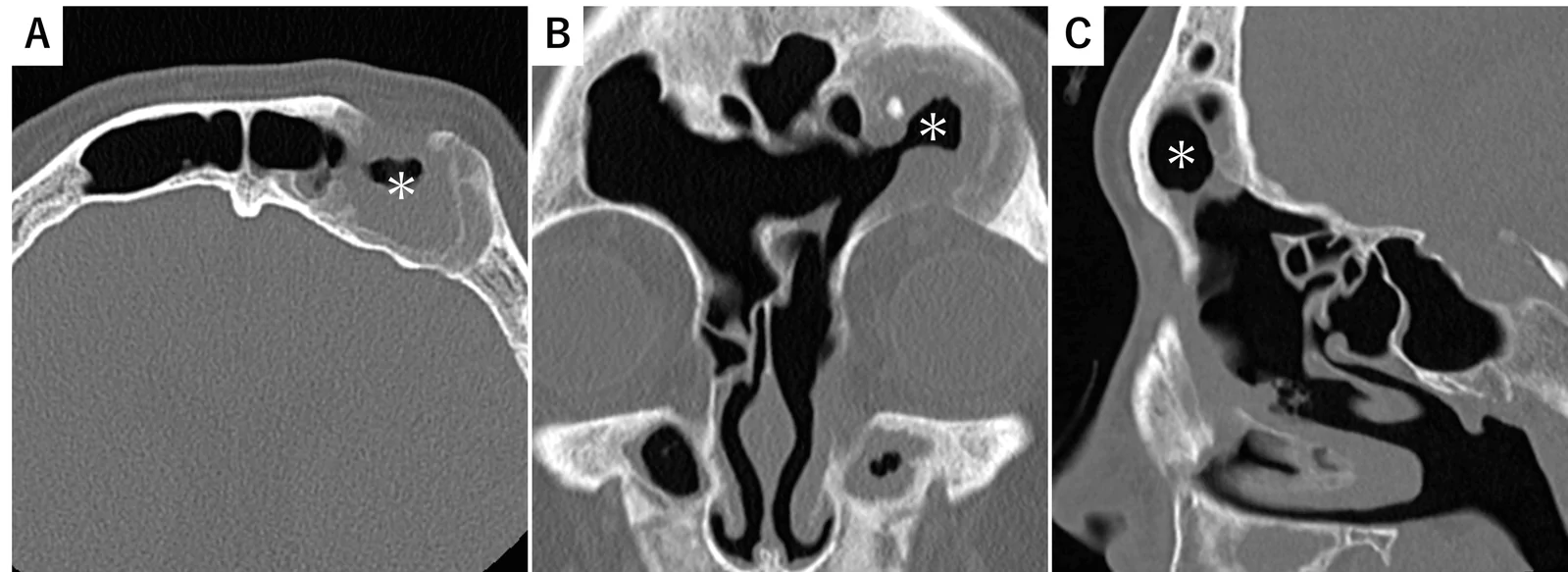
Computed tomography findings six months after surgery (A-C) Axial, coronal, and sagittal computed tomography images, respectively. The marsupialized cholesterol granuloma cavity had decreased in size, and the opening remained patent despite residual mucosal thickening. The surrounding bony walls had thickened, with reconstitution of the anterior frontal sinus wall and orbital roof at sites that had shown thinning and defects preoperatively. ^*^marsupialized cholesterol granuloma cavity

## Discussion

CG is a foreign-body granulomatous reaction to cholesterol crystals derived from the degradation products of blood components and most commonly occurs in the temporal bone. Although involvement of the paranasal sinuses is rare [1,2], the frontal sinus is the most frequently affected paranasal sinus. Combining the data reported by Durgam et al. [1] and Wershoven et al. [2], 91 of 153 cases (59.5%) involved the frontal sinus, followed by the maxillary sinus in 51 cases (33.3%), the ethmoid sinus in six (3.9%), and the sphenoid sinus in five (3.3%).

The proposed mechanisms of CG formation include chronic inflammation, impaired ventilation and drainage, and local hemorrhage [1,2]. Because the frontal sinus outflow tract is anatomically narrow and complex and is therefore prone to obstruction, this anatomy may partly account for the predominance of CG in the frontal sinus. In the present case, a small lesion suspected to be a mucocele was present medially within the frontal sinus. Narrowing or obstruction of the frontal sinus drainage pathway by this medial lesion may have contributed to the formation of the lateral CG, suggesting an important role for local impairment of ventilation and drainage in this patient.

CT and MRI are useful for the preoperative assessment of paranasal sinus CG. On CT, CG may appear as a well-circumscribed expansile lesion accompanied by thinning or defects of the surrounding bony walls. On MRI, blood degradation products frequently result in high signal intensity on both T1- and T2-weighted images. Among eight individual cases with reported MRI findings in the review by Durgam et al., six (75%) were hyperintense on T1-weighted images and five (62.5%) were hyperintense on T2-weighted images [1]. These imaging findings are not pathognomonic, and histopathological examination remains necessary for a definitive diagnosis. In the present case, the predominantly high signal intensity on both T1- and T2-weighted images and minimal contrast enhancement led to a preoperative suspicion of CG, which was confirmed by histopathological examination of the cyst wall.

Surgery is the mainstay of treatment for paranasal sinus CG. In the systematic review by Durgam et al. of 135 cases reported between 1970 and 2010, surgical approach data were available for 65 cases; 52 (80%) were managed using open procedures and 13 (20%) using endoscopic procedures [1]. Among the frontal sinus cases for which the specific procedure was reported, frontal orbitotomy was performed in 12, lateral orbitotomy in two, external frontoethmoidectomy in two, a bicoronal osteoplastic flap in one, and transfrontal craniotomy in one; all were open approaches [1]. Thus, open surgical management historically predominated in the treatment of frontal sinus CG.

Endoscopic treatment of frontal sinus CG began to appear in scattered reports during the 2010s. Marco et al. reported an open Lynch-Howarth procedure followed by an endoscopic frontal sinusotomy to restore and enlarge the frontal drainage pathway [5]. Deep et al. subsequently reported successful transnasal marsupialization using a Draf III procedure, supporting the feasibility of treatment using ESS alone [3]. More recently, Rustichelli et al. reported endoscopic treatment using a Draf IIb approach [6]. Nevertheless, reports of endoscopic treatment for frontal sinus CG remain limited.

A central consideration in selecting the surgical approach is whether to pursue complete excision or to perform marsupialization aimed at establishing drainage and ventilation. CG is a non-neoplastic inflammatory lesion associated with impaired ventilation and drainage and intracavitary hemorrhage. Therefore, when durable drainage and ventilation can be established, a treatment strategy that does not require complete excision of the cyst wall may be reasonable. Deep et al. reported successful Draf III marsupialization with mucosal grafting, with a patent cavity and no evidence of persistent CG at six months [3]. In contrast, Yokoi et al. described recurrence two months after initial ESS and emphasized complete removal of pathological mucosa during revision Draf III surgery, after which no recurrence was observed over five years [7]. However, because the revision procedure combined enlargement of the drainage pathway with mucosal removal, their respective contributions cannot be determined. These reports do not establish whether complete mucosal or cyst-wall excision is necessary in all cases. In the present case, although the lesion extended into the lateral frontal sinus, we judged that adequate endoscopic access could be achieved and selected endoscopic marsupialization rather than complete excision through an open approach as the preoperative strategy. We planned to first attempt access to the lateral frontal sinus using a Draf IIb approach and to convert to a Draf III procedure by adding a contralateral approach if manipulation proved difficult. Intraoperatively, the Draf IIb approach provided an adequate opening, and extension to a Draf III procedure was therefore unnecessary.

Draf IIb and III procedures may leave broad areas of exposed bone after drilling of the frontal sinus floor and frontal beak. Exposed bone can contribute to postoperative crusting, osteitis, scarring, and restenosis of the frontal neo-ostium. Omura et al. reported that the frontal neo-ostium remained patent throughout follow-up in all 19 patients who underwent Draf II or III procedures with coverage of exposed bone using a SLAP flap [4]. In the present case, the SLAP flap and a middle turbinate-based mucosal flap were used to cover exposed bone to promote early epithelialization and prevent restenosis. Postoperative narrowing may result from wound-healing changes, including scarring and neo-osteogenesis [4]. Persistent inflammation in retained tissue may also contribute, but its contribution cannot be determined from endoscopic appearance alone. In the present case, follow-up endoscopy showed an epithelialized cavity without retained contents, and CT demonstrated a reduction in cavity size with a patent opening despite residual mucosal thickening. These findings support maintained drainage but do not establish complete resolution of inflammation in the retained cyst wall.

A Penrose drain was placed to maintain patency of the marsupialized CG opening and facilitate postoperative irrigation. Frontal sinus stenting has been used in other clinical settings, but evidence of benefit is inconsistent. Weber et al. retrospectively reviewed 12 patients treated with six-month stenting and reported favorable outcomes [8]. In contrast, Banhiran et al. found no significant difference in ostial patency or symptom improvement with versus without short-term stenting after an endoscopic modified Lothrop procedure [9]. Khan et al. reported stent migration and foreign-body granulomatous tissue formation [10]. Their literature review also discussed secondary infection, hypersensitivity reactions, bacterial biofilm formation, and toxic shock syndrome associated with frontal sinus stents [10]. These reports do not directly establish the efficacy or optimal duration of Penrose drainage for frontal sinus CG. In this case, the drain was used for irrigation during outpatient follow-up and removed three months after surgery. No recurrence was observed during the subsequent 18 months. The drain may have contributed to early maintenance of patency, but its independent effect cannot be distinguished from that of wide marsupialization, mucosal flap coverage, and postoperative irrigation. The three-month placement period should therefore be regarded as the duration used in this case, rather than an established optimal interval. Drain placement and duration should be individualized, balancing the need to maintain drainage and irrigation access against potential device-related complications.

Among the 49 individual paranasal sinus CG cases with reported outcome data in the review by Durgam et al., recurrence occurred in four (8.2%), and the mean follow-up period was 34.5 months [1]. In the present case, the frontal sinusotomy and marsupialized opening remained patent without recurrence through 21 months after surgery. However, this follow-up period remains shorter than that reported in the review, and continued long-term endoscopic and radiologic surveillance is necessary to detect restenosis of the opening or re-expansion of the lesion.

Although reports of endoscopic treatment for frontal sinus CG remain limited, endoscopic marsupialization can be an effective, minimally invasive alternative to an open approach when adequate access can be achieved. Because access to lesions in the lateral frontal sinus may be difficult, the surgical approach should be individualized according to lesion location, the degree of frontal sinus pneumatization and other anatomic factors, and the surgeon's experience.

## Conclusions

Frontal sinus CG is a rare lesion that has traditionally been managed by open surgical approaches. In the present case, a Draf IIb approach provided adequate access to a laterally extending lesion, allowing wide endoscopic marsupialization while avoiding an external incision. Endoscopic marsupialization is a useful, minimally invasive treatment option when lesion location and frontal sinus anatomy permit adequate access. Careful long-term endoscopic and radiologic follow-up remains necessary.
